# General cross-modality registration framework for visible and infrared UAV target image registration

**DOI:** 10.1038/s41598-023-39863-3

**Published:** 2023-08-09

**Authors:** Yu Luo, Hao Cha, Lei Zuo, Peng Cheng, Qing Zhao

**Affiliations:** College of Electronic Engineering, Naval University of Engineering, Wuhan, 4300000 China

**Keywords:** Electrical and electronic engineering, Applied optics, Optical techniques, Information technology

## Abstract

In all-day-all-weather tasks, well-aligned multi-modality images pairs can provide extensive complementary information for image-guided UAV target detection. However, multi-modality images in real scenarios are often misaligned, and images registration is extremely difficult due to spatial deformation and the difficulty narrowing cross-modality discrepancy. To better overcome the obstacle, in this paper, we construct a General Cross-Modality Registration (GCMR) Framework, which explores generation registration pattern to simplify the cross-modality image registration into a easier mono-modality image registration with an Image Cross-Modality Translation Network (ICMTN) module and a Multi-level Residual Dense Registration Network (MRDRN). Specifically, ICMTN module is used to generate a pseudo infrared image taking a visible image as input and correct the distortion of structural information during the translation of image modalities. Benefiting from the favorable geometry correct ability of the ICMTN, we further employs MRDRN module which can fully extract and exploit the mutual information of misaligned images to better registered Visible and Infrared image in a mono-modality setting. We evaluate five variants of our approach on the public Anti-UAV datasets. The extensive experimental results demonstrate that the proposed architecture achieves state-of-the-art performance.

## Introduction

**Figure 1:**
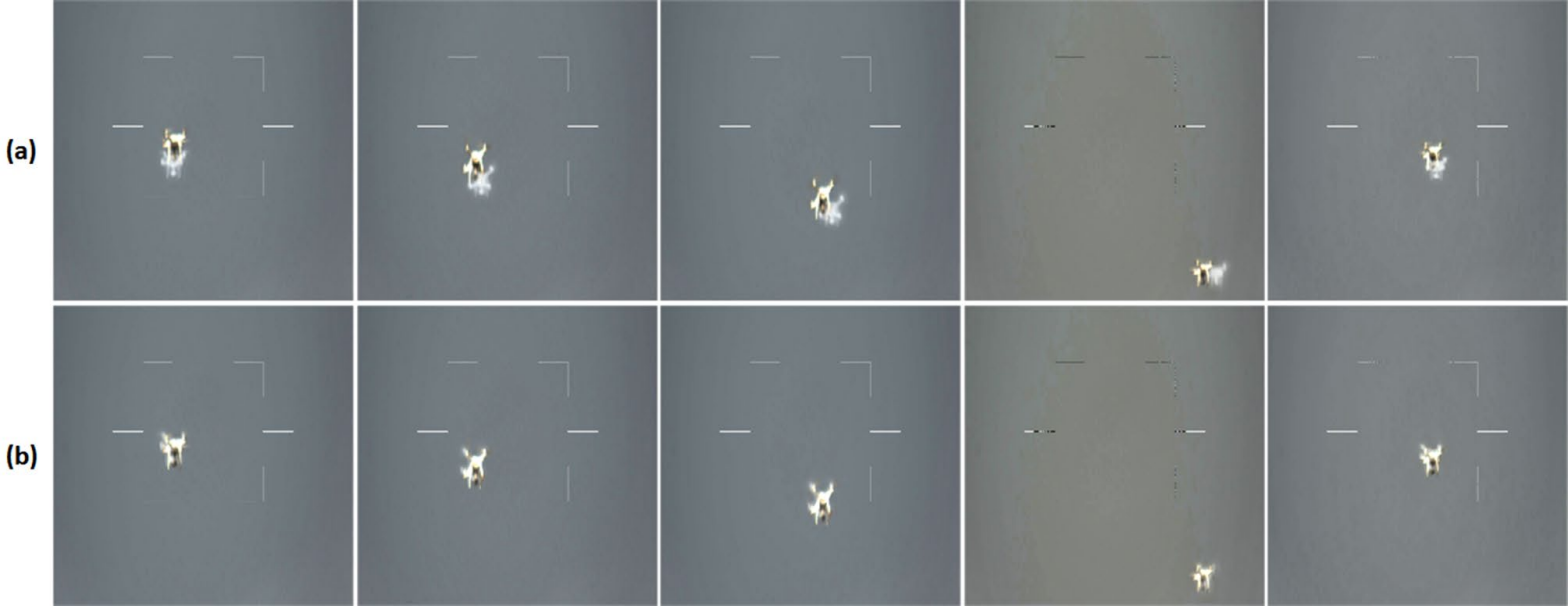
An example of UAV target images misalignment and alignment. To elaborate more clearly the problem of misalignment between cross-modal images, we fused the VIS and IR images using the PIAFusion^1^ algorithm, and the fused results are shown in the figure above. (**a**) Fusion results of direct registration of misaligned cross-modal images. Such fused images is often accompanied by severe artifacts, which can significantly affect downstream work. (**b**) Fusion results after cross-modal registration using the algorithm proposed in this paper. In contrast, the proposed registration method shows desirable alignment and ghost elimination.

With the significant surged in accessibility and popularity of Unmanned Aerial Vehicles (UAVs), the phenomenon of black flying is also becoming increasingly serious, and the dangers it brings are enormous. Behind these potential threats, monitoring the operational status of drones is crucial, including detection and tracking.

In recent research, most of UAV target detectors and trackers are based on Visible (VIS) information^2–6^. When in the adverse weather and low light conditions these trackers might not be able to find useful cues, leading to unreliable results. Therefore, some works consider using fuse information from VIS and Infrared (IR) images for object detection and tracking^7,8^ in order to accomplish UAV target detection and tracking tasks under all-day-all-weather requirements. However, within the existing literature, the majority VIS and IR images fusion methods^9–11^ perform well only under images well-alignment conditions, but fail under conditions of images misalignment. The intrinsic reason is that existing image fusion methods are sensitive to differences in intensity between spatially misaligned VIS and IR images and can produce severe ghosting artifacts on the fused images once there is a slight offset and distortion (see Fig. 1a). In these cases, cross-modal aligned image (see Fig. 1b) are essential for proper execution of the aforementioned downstream tasks.

After years of research, many methods have been proposed to attempt to solve the challenge to Cross-Modality images registration, which can be broadly divided into two categories: feature-based registration and learning-based registration. The feature-based methods typically consist three steps: feature extraction and description, feature matching, estimation of translation model parameters. Due to the severe nonlinear intensity differences between infrared and visible images, traditional feature matching descriptor such as SIFT^12^, ORB^13^, and SURF^14^ perform poorly under multimodal conditions.

The learning-based registration methods performs pixel-level and feature-level alignment by directly estimating the distortion field between the distorted image and its reference image^15,16^. Such algorithms for direct estimation of deformation fields, while well suited to unimodal registration problems, still perform poorly in a multimodal settings^17^. Given the recent success of multimodal image translation^18,19^, the researcher began to consider using cross-modal translation networks to convert the multi-modality registration problem to a simpler unimodal alignment problem. Specifically, the cross-modal translation networks uses a generative adversarial network (GAN) model to transform the image from the source modality to the target modality.

**Figure 2:**
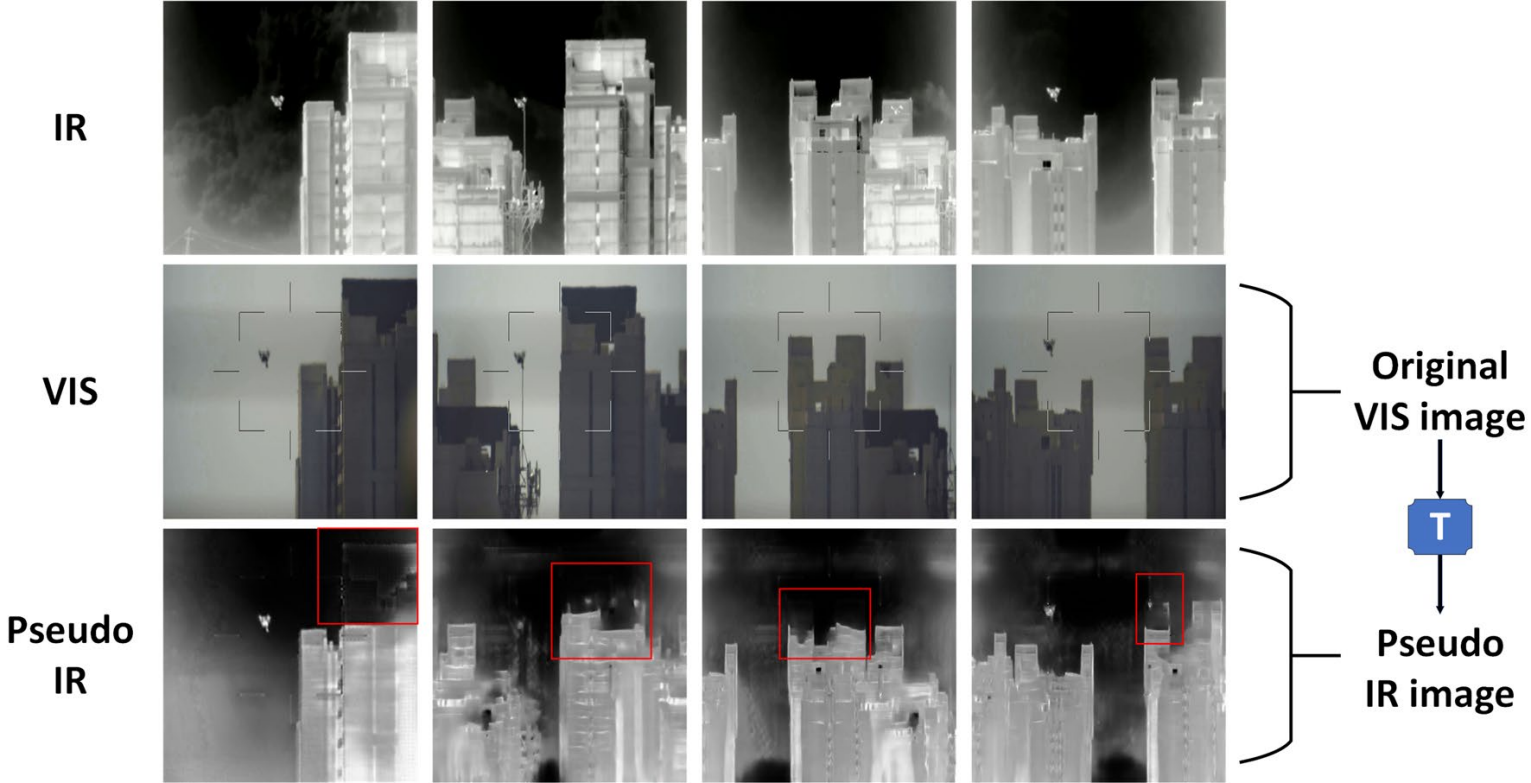
Illustration of shape inconsistencies. The first and second rows in the figure above represent the original IR image and the VIS image respectively. *T* in the figure indicates the modal translation network based on the CycleGAN^20^. The last row represent the pseudo IR image obtained from the VIS image by the modal translation network *T*. By comparing the original IR with the pseudo IR image, it is clear that the pseudo IR image produces severe shape inconsistencies in the area marked by the red box.

Nevertheless, such GAN-based image translation tends to produce shape inconsistencies, which in turn worsen the performance of registration^21^. More specifically, Chen et al.^22^ contended that the inconsistency and artifacts are introduced by the discriminator that mistakenly encodes domain-specific deformations as indispensable appearance features and encourages the generator to reproduce the deformations. This tends to create unnecessary difficulty for registration tasks. As shown in the last row of Fig. 2, very serious shape inconsistencies occur when we use GAN-based approach to translate VIS images to pseudo-IR images.

To address the deterioration in registration caused by shape inconsistencies, Casamitjana et al.^23^ presents SbR, which introduce a registration loss for weakly supervised image translation between domains that does not require perfectly aligned training data. This loss capitalises on a registration U-Net with frozen weights, to drive a synthesis CNN towards the desired translation. SbR complement this loss with a structure preserving constraint based on contrastive learning, which prevents blurring and content shifts due to overfitting. Despite this, their registration network is first trained on the images from the target modality instead of images from the two modalities, which may guide the registration network to generate an unrealistic deformation field. This unrealistic deformation field will result in a loss of registration accuracy.

Furthermore, we also found that if only simply upgrading the geometry preservation capability of the modal transfer network would not yield the best registration accuracy for UAV targets. This is explained by the fact that the size of the UAV target is much smaller than the background size and the texture feature information is not sufficient, which leads to small targets being ignored during the registration process.

It is not so hard to reveal the reasons of the above dilemma posed to the small targets registration. Digging into the details of current cross-modality registration method, it can be easily figured out that the designed registration method focuses more on improving the alignment accuracy of global features, while ignoring the issue of local alignment. These registration methods do not fully exploit existing feature information and reuse it, which leaves the low-level patterns of images at shallow network layers unexplored and the small-scale details are dismissed. Thus the parameters for registration networks may focus little on small-scale textures and are dominated by Large-scale semantics. This is fatal to the UAV target registration task studied in this paper.

Motivated by lessons learned through the above analysis, we attempt to address these issues in terms of both enhancing the geometry preservation capability of the modal transfer network and the small-scale feature extraction and reuse capability of the registration network.

In this work, we present a novel unsupervised framework named General Cross-Modality Registration (GCMR) for multi-modality registration. GCMR is able to accurately complete modal transfer and obtain sufficient feature information for registration of smaller UAV targets. Specifically, we have designed a plug-and-play Structure Correction Network (SCN) for enhancing the geometry preservation capability during translation. The presented SCN incorporates a perceptual loss and a adversarial loss to integrate the output with the target geometry and appearance. Additionally, content loss is also applied to the SCN and its coefficient is set very large, with the aim of forcing the network to maintain shape consistency during translation. We have also designed a Multi-level Residual Dense Registration Network (MRDRN) for boosting the small-scale feature extraction and reuse capability of the registration network. The presented MRDRN combined with residual and dense connection structures. This structured network not only achieves basic global alignment, but also captures detailed local texture information by modelling detailed image patches to drive alignment of small targets. The proposed SCN coupled with this MRDRN can achieve local and global alignment and yield more accurate deformation fields.

The main contributions of our work are:

- We present an novel unsupervised VIS-IR Image registration model that effectively and accurately achieves rigid alignment of UAV targets in complex background. As far as we know, this is the first time to attempt to extend the original cross-modality generation-registration paradigm to the field of UAV target detection.
- We design a Structure Correction Network (SCN) that make translation network has stronger geometry structure preservation capability and allows for better application of mono-modality metrics in multimodal registration.
- We also design a Multi-level Residual Dense Registration Network (MRDRN) to further improve registration performance, especially for small UAV targets.

## Related work

With the rapid development of deep learning technology, the effectiveness of feature based registration methods has fallen far behind that of learning based registration methods. Therefore, in recent work, researchers have preferred the learning-based registration approach.

Balakrishnan et al.^16^ proposed the VoxelMorph model, that relies on a CNN network and a spatial translation layer and smoothing constraints on the deformation field, with the aim of training a parametric function to perform direct alignment on new input image pairs. The proposed method is unsupervised and does not need standard alignment images and anatomical labels. This direct alignment of various modal images does not take into account the variations in optical features, geometric features and spatial locations expressed within the infrared and visible images, therefore the results are not adequate.

To overcome this barrier to registration caused by variations in modal information, Wei et al.^24^ proposed a gradient guided multispectral image registration model, known as RegiNet. RegiNet uses the gradient map of the reference image to guide the target image for alignment, to compensate for feature intensity inconsistencies between visible and infrared images, and to facilitate the network ability to better align image edges. Qin et al.^25^ use image disentanglement to decompose images into common domain-invariant latent shape features and domain-specific appearance features. Then the latent shape features of both modalities are used to train a registration network. Arar et al.^19^ attempt to bypass the difficulties of developing cross-modality similarity measures, by training an image-to-image translation network on the two input modalities. This learned translation allows training the registration network using simple and reliable mono-modality metrics. Chen et al.^25^ combines adversarial loss with similarity measures to correctly register the images, while focusing on preserving local geometric properties. They encode the inputs into two separate embedding, one for shape and one for content information, and train a registration network on these disentangled embedding. This method relies on learned disentanglement, which introduces inconsistencies at the local level.

To further solutions to the cross-modal registration challenge, researchers began to contemplate the translation of the multimodal alignment problem into a mono-modality alignment problem by means of modal translation and proposed the cross-modal generation-registration paradigm. Chen et al.^22^ approach combines a discriminator-free translation network to facilitate the training of the registration network, and a patchwise contrastive loss to encourage the translation network to preserve the shape of the object. In addition, the method proposes to replace the adversarial loss widely used in previous multimodal image alignment methods with a pixel loss in order to integrate the output of panning into the target modality. Wang et al.^17^ propose a Crossmodality Perceptual Style Transfer Network to generate a pseudo infrared image. The generated pseudo infrared image embraces a sharp structure, which is more conducive to transforming cross-modality image alignment into mono-modality registration coupled with the structure-sensitive of the infrared image. All of the these methods rely on cycle consistency and GAN mode during the modal transition phase.

However, cycle consistency leads to multiple solutions, which means that the translated images can not maintain the structure consistency of source images and may contain artifacts^26^. On the contrary, our proposes a simple and efficient structure correction module that fundamentally solves the multiple solutions issue.

## Methods

**Figure 3:**
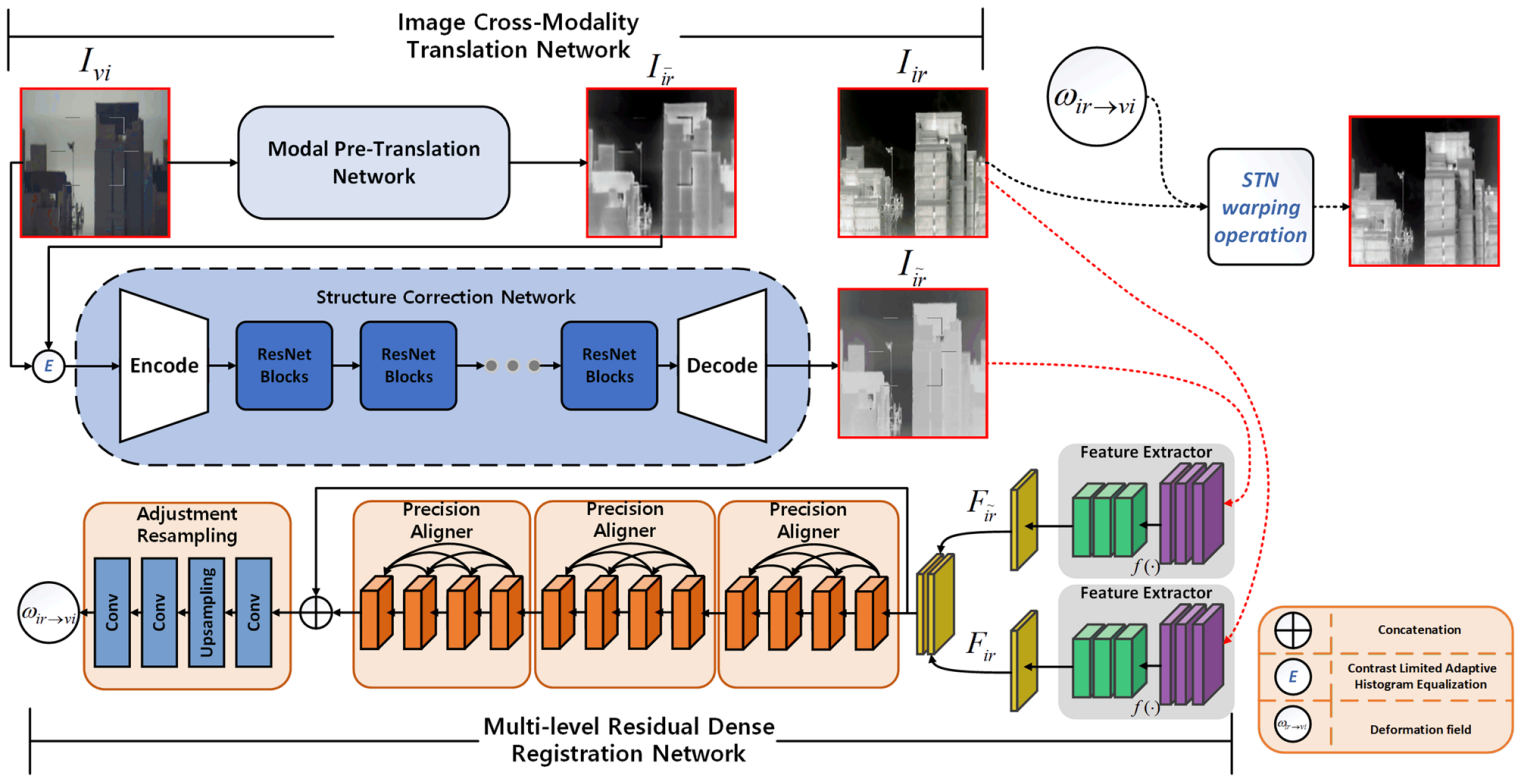
The pipeline of the GCMR Framework. Our model consists of two sub-networks, which are Image Cross-modality translation Networkand a Multi-level Residual Dense Registration Network. Our proposed model takes misaligned infrared and visible images as input, and then executes the above two sub-networks in turn to obtain the final well-aligned images.

In this section, we will introduce the proposed cross-modality generation registration model in detail. The entire structure consists of two modules, a Image Cross-Modality Translation Network (ICMTN) *T*() and a Multi-level Residual Dense Registration Network (MRDRN) *R*(), shown in Fig. 3. The ICMTN is built on Style Transfer model, which for mapping images from source domain to target domain and reconstructing images from the target domain. After generating the imitation pseudo-infrared image using ICMTN, we used MRDRN to enable the infrared image to be aligned with it in the spatial domain. Our proposed model follows the specialized cross-modality generation-registration paradigm^17^ and aims to reduce spatial offsets and alleviate the ghost during misaligned infrared and visible image registration.

### Image cross-modality translation network

The Image Cross-Modality Translation Network (ICMTN) *T*() is a two-stage model which objective lies in the mapping of the image from the source domain to the target domain and the reconstruction image from the target domain. In the first stage, we use the Modal Pre-Translation Network (MPTN) *R*() to generate rough pseudo-infrared images $I_{\tilde{ir}}$. The aim of MPTN is to reduce cross-modal discrepancies between modalities, allowing difficult multimodal registration problems to be converted to simple unimodal registration problems. Network structure of MPTN following with CycleGAN^20^, except that we have replaced its original discriminator with U-Net^27^ and reduced the frequency of discriminator updates. Mathematically, MTPN is described as,

1 $$\begin{aligned} {I_{\bar{ir}}} = T_{\theta }^{pre}(I_{vi}) \quad ( I_{vi},{I_{\bar{ir}}} \in R^{H\times {W}}). \end{aligned}$$

Where $T_{\theta }^{pre}$ denotes the MPTN with network parameter $\theta$. With the MPTN , the multi-modality registration task is converted into a unimodal one. However, In the task of modal converting visible to pseudo infrared images, the existing network model leads to multiple solutions, which means that the translated images can not maintain the geometric structure of source images and may contain artifacts and shape inconsistencies^26^. Such multiple solutions tend to in worsen the performance of registration.

In this paper, we have tendency solve the matter with a new perspective. We propose a Structure Correction Network (SCN) employed in the second stage, that is used to modify the structural information bias of the generated pseudo infrared images. The core idea of SCN is to reuse the clear structural information of the original VIS image and to train it in combination with content loss. The aim is to force the generated pseudo-infrared images to have an accurate modal pattern while obtaining a unambiguous geometric structure. The use of SCN has been shown to be effective in avoiding the multiple solutions problem.

**Figure 4:**
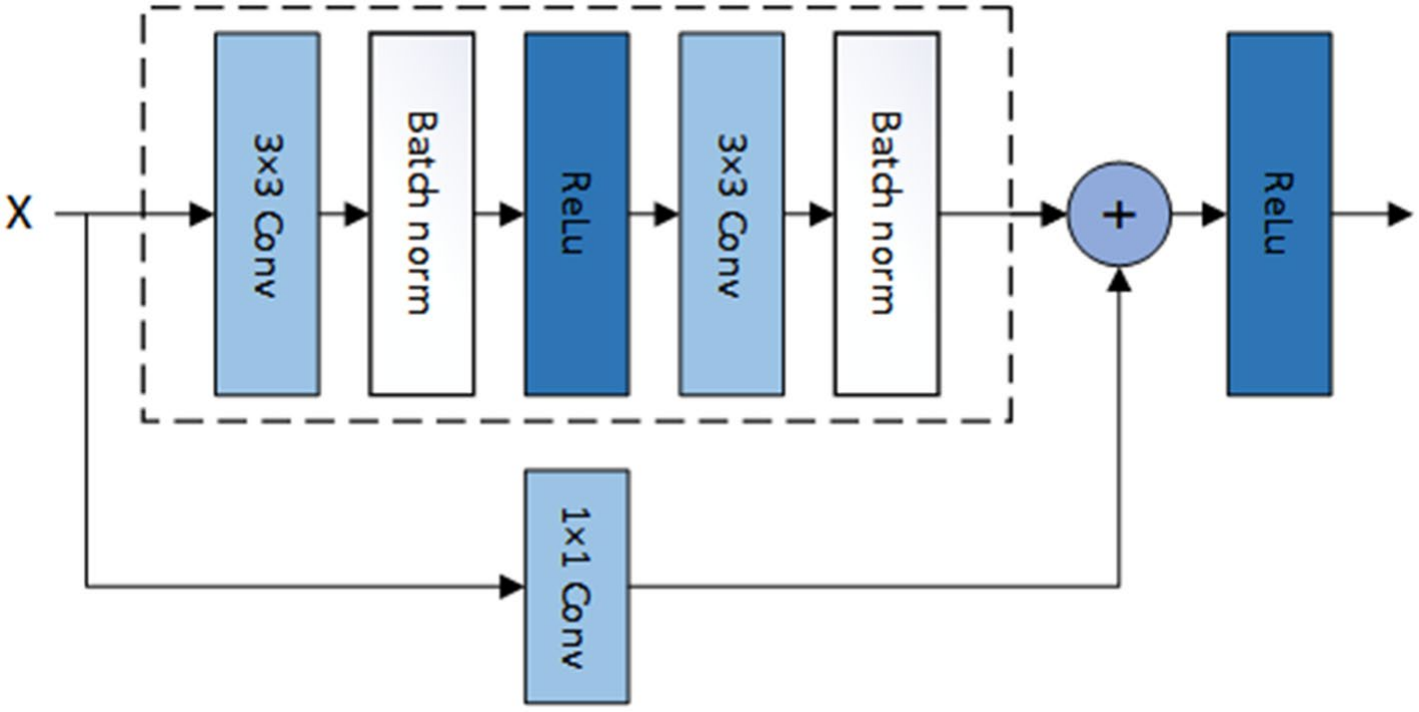
The ResNet Blocks consists of two dense layers and a skip connection. The activation function of each two dense layers are relu function. Batch norm layers normalize the features using mean and variance in a batch during training and use estimated mean and variance of the whole training datasets during testing.

Specifically, for pre-processing, We get a texture-enhanced image $I_{enh}$ as the input to the SCN. $I_{enh}$ can be calculated as $I_{enh}={{I_{\bar{ir}}}\oplus { E(Gray(I_{vi}))}}$, Where $I_{\bar{ir}}$ is the pseudo-infrared image generated by MPTN. *Gray*() denotes graying of the image and is for Contrast Limited Adaptive Histogram Equalization (CLAHE) algorithm, which is used to enhance the edge information of the image. The architecture of SCN is shown in Fig. 3, which includes an encoder $T_{enc}^{SC}$, seven ResNet Blocks modules $T_{res}^{SC}$ and a decoder $T_{dec}^{SC}$. The network structure of ResNet Blocks shown in Fig. 4.

$T_{enc}^{SC}$ extracts shape-related features multiple granularity, then use $T_{res}^{SC}$ to fully evaluate and exploit low-level patterns, while $T_{dec}^{SC}$ learns to perform shape preserving modality translation with those features. Given the input $I_{enh}$, $T_{enc}^{SC}$, $T_{res}^{SC}$ and $T_{dec}^{SC}$jointly generate the output $I_{\tilde{ir}}$ which can be calculated as:

2 $$\begin{aligned} {I_{\tilde{ir}}}={T_{\theta }^{SC}(I_{enh})}=T_{dec}^{SC}{(T_{res}^{SC}{(T_{enc}^{SC}{(I_{enh})})})}. \end{aligned}$$

Where $I_{\tilde{ir}}$ is the pseudo-infrared image corrected for structural information. Note that the SCN is designed as a simple yet effective GAN architecture[40]. $T_{\theta }^{SC}$ corresponds to the generator in our SCN. In summary, our full objective of ICNT is:

3 $$\begin{aligned} {I_{\tilde{ir}}}={T_{\theta }^{SC}({T_{\theta }^{pre}}(I_{vi}))}. \end{aligned}$$

Further, we introduce a Relativistic discriminator to help SCN to capture fine Structure Information under adversarial learning setting. We define the Relativistic discriminator *D*() following ESRGAN^28^.

### Multi-level residual dense registration network

Since ICMTN reduces cross-modal discrepancy, inter-image alignment becomes a unimodal task and alignment is much less difficult. In order to further improve the alignment capability of the registration network, we exploit a Multi-level Residual Dense Registration Network (MRDRN). MRDRN *R*() takes an image pairas $(I_{ir},I_{\tilde{ir}})$ an input and outputs a deformation field $\omega =R(I_{ir},{I_{\tilde{ir}}})$. The warped imageis $I_{ir}^{reg}$aligned with $I_{\tilde{ir}}$. In a two-dimensional setting, the deformation field is a matrix of 2D vectors, indicating the moving direction for every pixel in the source image $I_{ir}$.

**Figure 5:**
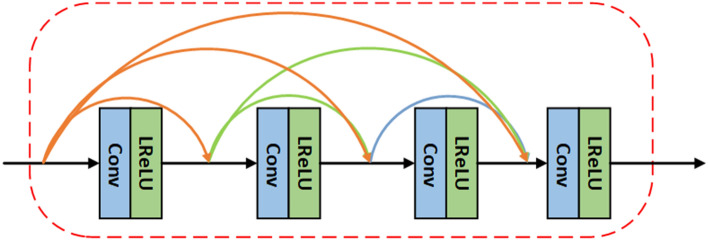
Structure of Precision Aligner. The Precision Aligner combines multi-level residual network and dense connections. This network structure can fully extract and exploit the small-scale feature information in the image, which can better serve the requirements of small UAV target registration tasks. Moreover, Batch Norm layers is removed from the convolution block, an operation that has been proven to increase performance and reduce computational complexity^28^.

The MRDRN consists of a Feature Extractor, three Precision Aligner and a Adjustment Resampling module. Where the Feature Extractor acquires the feature map from the image pair $(I_{ir},I_{\tilde{ir}})$ and then feeds it to the first Precision Aligner (Structure of Precision Aligner is shown in Fig. 5) and outputs an alignment matrix $\delta ^{1}$.

The mathematical expression for $\delta ^{1}$ can be described as:

4 $$\begin{aligned} \delta ^{1}=(A^1{({f(I_{ir},I_{\tilde{ir}}})})+f{(I_{ir},{I_{\tilde{ir}}}})). \end{aligned}$$

With repeated utilization of Precision Aligner the contour information of the image is gradually aligned and the mathematical process is described as:

5 $$\begin{aligned} \delta ^{k}={A^{k}}({\delta ^{k-1}})+{\delta ^{k-1}} \quad k\in (2,3). \end{aligned}$$

Once $\delta ^{k}$ has been obtained, it is fed into the Adjustment Resampling module. Adjustment Resampling module consists of a convolutional block, an upsampling block and three other convolutional blocks in sequence, which is to convert the $\delta ^{k}$ obtained from the Precision Aligner into a conveniently usable deformation field $\omega$:

6 $$\begin{aligned} \omega =R({(I_{ir},I_{\tilde{ir}}})=adjust(\delta ^{k-1}). \end{aligned}$$

Finally, we obtain an reconstruct the registered infrared image by employing the warping operation algorithm:

7 $$\begin{aligned} I_{ir}^{reg}=Warp({I_{ir},\omega }). \end{aligned}$$

Where warping operation *Warp*() is based on STN^29^.

### Loss function

During training, the MPTN and the SCN are both optimized with adversarial loss[6], while the SCN further adopts content loss and perceptual loss. Thus, we first describe MPTN loss $L_{MPTN}$ and SCN $L_{SC}$ loss separately, and then introduce the full objective of two networks.

#### Loss of modal pre-translation network

MPTN is similar to the CycleGAN^20^ design, which apply adversarial losses to both mapping functions. For the mapping function $G_{MPTN}:{I_{vi}\rightarrow {I_{ir}}}$ and its discriminator $D_{MPTN}^{I_{ir}}$, we express the objective as:

8 $$\begin{aligned} {L_{GAN}(G_{MPTN},D_{MPTN}^{I_{ir}},I_{vi},I_{ir})}=E_{I_{ir}}[{\log {D_{MPTN}^{I_{ir}}(I_{ir})}]+E_{I_{vi}}}[{\log ({1-D_{MPTN}^{I_{ir}}(G(I_{ir})))}}]. \end{aligned}$$

Similarly, adversarial loss for the mapping function and $F_{MPTN}:I_{ir}\rightarrow I_{vi}$ its discriminator $D_{MPTN}^{I_{vi}}$ as well:

9 $$\begin{aligned} L_{GAN}(F_{MPTN},D_{MPTN}^{I_{vi}},I_{ir},I_{vi}). \end{aligned}$$

To further reduce the space of possible mapping functions, the cycle consistency loss is introduced:

10 $$\begin{aligned} L_{cyc}(F_{MPTN},F_{MPTN})=E_{I_{vi}}[\Vert {F_{MPTN}(G_{MPTN}(I_{vi}))-I_{vi}}\Vert _1]+E_{I_{ir}}[\Vert {G_{MPTN}(F_{MPTN}(I_{ir}))-I_{vi}}\Vert _1]. \end{aligned}$$

Our full objective of MPTN is:

11 $$\begin{aligned} L_{MPTN}={L_{GAN}(G_{MPTN},D_{MPTN}^{I_{ir}},I_{vi},I_{ir})}+L_{GAN}(F_{MPTN},D_{MPTN}^{I_{vi}},I_{ir},I_{vi})+\lambda _{cyc}L_{cyc}(G_{MPTN},F_{MPTN}). \end{aligned}$$

Where $\lambda _{cyc}$ controls the relative importance of the two objectives.

#### Loss of structure correction network

In training phase of SCN, the parameters of MPTN are fixed and the training loss is built upon ${I_{\bar{ir}}}$. To better learn sharper edges and more detailed textures, we introduce a Structural Correction loss to control train of SCN. The Structural Correction loss $L_{SC}$ consists of three terms known as perceptual loss $L_{percep}$, adversarial loss $L_{adc}$ and Content Loss $L_{con}$. First, the $L_{percep}$ is defined as:

12 $$\begin{aligned} L_{percep}=\frac{1}{W_{i,j}H_{i,j}}{\sum _{x=1}^{W_{i,j}}} \sum _{y=1}^{H_{i,j}}(\phi _{i,j}(I_{ir})_{x,y}-\phi _{i,j}(G_{SC}(I_{enh}))_{x,y})^2. \end{aligned}$$

Where $\phi _{i,j}$ indicate the feature map obtained by the $j$-th convolution before the $i$-th maxpooling layer within the VGG19[42] network, $W_{i,j}$and $H_{i,j}$ describe the dimensions of the respective feature maps within the VGG network.

      The adversarial loss for generator is in a symmetrical form:

13 $$\begin{aligned} L_{adv}=-E_{I_{ir}}[\log (1-D_{SC}(I_{ir},I_{enh}))]-E_{I_{enh}}[\log (D_{SC}(I_{enh},I_{ir}))]. \end{aligned}$$

In addition to the base perceptual loss and adversarial loss, we also introduce content Loss. The Content loss aims to correct the structural information of rough pseudo-infrared images via reducing the structured information difference between $I_{vi}$ and $I_{enh}$. Content loss is defined as:

14 $$\begin{aligned} L_{con}=E_{I_{enh}}{\Vert G_{SC}(I_{enh})-I_{vi} \Vert _1}. \end{aligned}$$

Where $L_{con}$ evaluate the 1-norm distance between recovered image $G_{SC}(I_{enh})$ and the ground-truth $I_{vi}$.

Therefore, The overall optimization objective of SCN is defined as:

15 $$\begin{aligned} L_{SC}=L_{percep}+\lambda _{adv}L_{adv}+\lambda _{con}L_{con}. \end{aligned}$$

$\lambda _{adv}$ and $\lambda _{con}$ are the coefficients to balance different loss terms.

#### Loss of multi-level residual dense registration network

To enable *R*() to learn the alignment at the global level, we formulate a popular registration loss function $L_{Rg}$, which consists of two components $L_{sim}^{bid}$ and $L_{smooth}$. We leverage bidirectional structural similarity loss similar to the UMF-CMGR^17^ to constrain the registration between distorted and pseudo infrared images in feature space, which is defined as

16 $$\begin{aligned} {L_{sim}^{bid}=\Vert {I_{ir}^{reg}-I_{\tilde{ir} }\Vert _1+\lambda _{rev}\Vert {Warp(I_{\tilde{ir}},-\omega )-I_{ir}}\Vert _1}}. \end{aligned}$$

Where $\lambda _{rev}$ is a regularization parameter.

Minimizing $L_{sim}^{bid}$ will encourage $I_{ir}^{reg}$ to approximate $I_{\tilde{ir}}$, but may generate a discontinuous $\omega$. We encourage a smooth $\omega$ using a diffusion regularizer on its spatial gradients:

17 $$\begin{aligned} L_{smooth}=\Vert {\nabla \omega }\Vert ^2. \end{aligned}$$

The complete loss is therefore:

18 $$\begin{aligned} L_{Reg}=\lambda _{sm}L_{smooth}+L_{sim}. \end{aligned}$$

Where $L_{sm}$ is a smooth parameter.

#### Final objective

Our final objective is as follows:

19 $$\begin{aligned} L_{total}=L_{MPTN}+L_{SC}+L_{Rg}. \end{aligned}$$

We train our network by minimizing the above total loss function to achieve the registration between infrared and visible images.

## Experiments

In the following section, comprehensive experiments are performed to demonstrate the generalization performance and robustness of our GCMR. Firstly, to demonstrate the wide generalization ability, we integrate our GCMR framework with five recent SOTA cross-modal registration models, and test them on Anti-UAV^30^. Secondly, several ablation studies are conducted to verify the effectiveness of each module of our GCMR.

### Experiments settings

#### Datasets details

Anti-UAV is the first UAV multimodal tracking datasets. Anti-UAV datasets includes six UAV types, two light modes (IR and VIS) and various backgrounds, stored in mp4 format and at a frame rate of 25 fps. Open source Anti-UAV datasets with 100 pairs of available video data. To meet the training requirements of the network, we extracted the video data into frames and resize the images to 256 × 256. Due to the high similarity of the cropped data, 2750 image pairs with different scenes were selected as the datasets. We randomly select 70% image pairs for training and 30% image pairs for testing. In addition, the image data for the two modalities in this datasets are not pre-aligned, witch is extremely unfriendly to downstream detection or tracking tasks.

#### Implement details

Our model are implemented in PyTorch and all the experiments were conducted on GeForce RTX 2080 Ti. We use Adam optimizer to train our model for 1200 epochs with parameters $lr=0.0003$, $\lambda _{adv}=0.8$, $\lambda _{con}=1.1$, $\lambda _{rev}=0.2$ and $\lambda _{sm}=0.2$. Linear learning rate decay is activated after 800 epochs.

#### Metrics

For the Anti-UAV datasets, we directly use VIS and IR images to evaluate the registration accuracy. We evaluate the registered results using six common metrics including NCC^31^, SSIM^32^, HIST^33^, PSNR^34^, NMI^35^ and MSE^36^. A higher NCC, SSIM, HIST, PSNR, NMI and lower MSE indicate a better performance of the registration model.

#### Baselines

We compare our method against five recent state-of-the-art multi-modality registration methods and some other well-established methods. Specifically, the competing methods are: SbR^23^, DFMRI^22^, UMF-CMGR^17^, NEMAR^19^, VoxelMorph^16^. For a fair comparison, we use either their publicly available codes or the implementations with recommended parameter settings. All methods are retrained on the Anti-UAV training datasets.

### Comparison with the state-of-the-arts

#### Modal translation analysis

**Figure 6:**
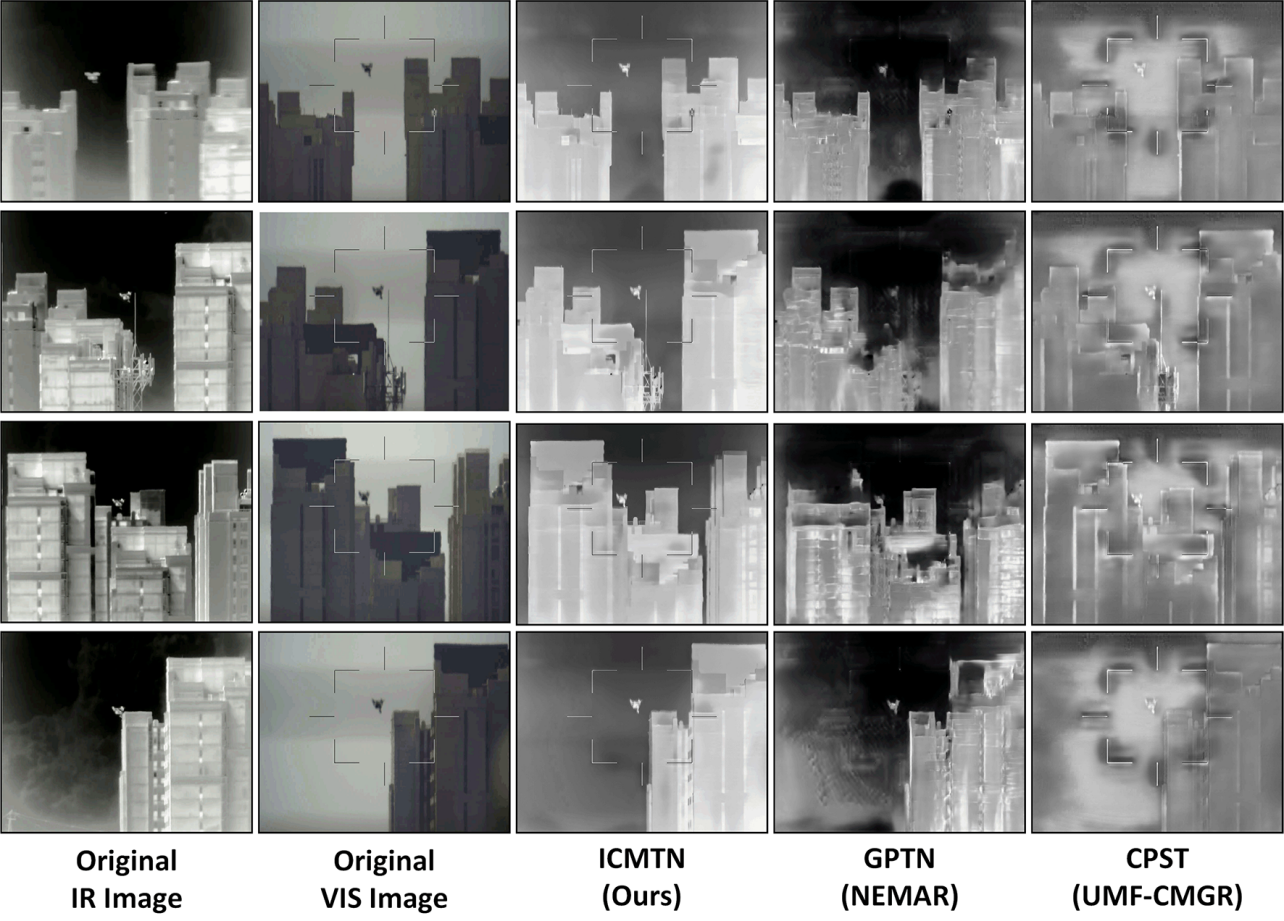
Visualization results of our Cross-modal translation method against other methods. The original IR and VIS image is shown in column 1–2. We show the cross-modal transfer results for three methods in columns 3–5: our method, GPTN^19^ (Using in NEMAR) and CPST^17^ (Using in UMF-CMGR). GPTN and CPST is the most recent state-of-the-art methods. It is worth mentioning that the modal translation of DFMIR and SbR fail, so the results are not shown.

We analyze the impact of using different cross-modal transfer models. As shown in Fig. 6, with using the GPTN, the generated pseudo infrared image suffers grossly structural degradation and the UAV target is barely visible (Fourth column) compared with the reference image (First column). Using CPST, the model retains general structural information, while subtle structures are not maintained well enough and “Blur” are introduced obviously (Fifth column). Moreover, CPST does not translate modalities well, and incorrect foreground and background modal information instead leads to a significant reduction in registration. In contrast, the pseudo infrared image generated by our model (Third column) has a sharper geometry structure, which caters to the common sense that infrared image “emphasizes structure over texture”.

It is apparent that the ICMTN proposed in this paper is better able to perform cross-modal transition tasks and effectively improves geometry preservation. At the same time, the pseudo-infrared images with clear geometric structure generated by ICMTN can facilitate the training of the registered network.

#### Evaluation on the anti-UAV test set

**Figure 7:**
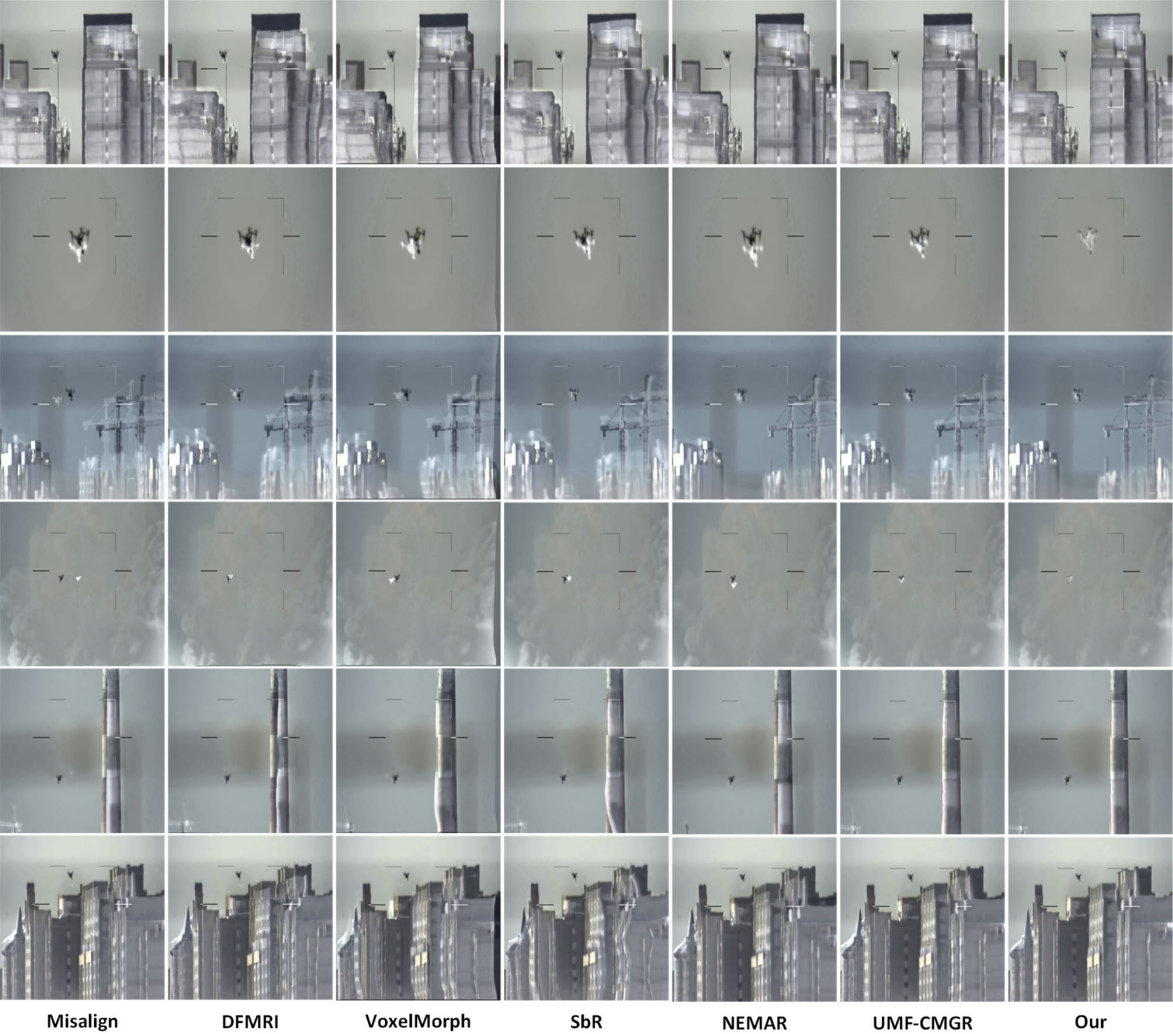
Visualization results of our method against other methods. The misaligned image is shown in column 1. We show the registration results of six methods: DFMRI, VoxelMorph, SbR, NEMAR, UMF-CMGR and Ours. Each registration results occupies one columns. VoxelMorph is the most basic unimodal registration network and remaining four methods are the most recent state-of-the-art methods for cross-modal registration issues.

The primary objective of our work is to achieve accurate multi-modality registration. The quantitative registration results on the Anti-UAV test set are summarized in Table 1. We can see that our method outperforms all the other state-of-the-art methods on all six metrics. Figure 7 shows the qualitative comparison of our method with the others. With using the DFMIR, VoxelMorph and SbR, the generated alignment image contains some distorted artefacts. This explained by the fact that these three types of algorithms do not perform cross-modal transitions or fail to perform cross-modal translation, thus preventing the registration network from finding a suitable reference target for alignment. NEMAR and UMF-CMGR produces relatively accurate alignment accuracy but still poor results. This is because the cross-modal translation network of NEMAR and UMF-CMGR rely on cycle consistency and GAN mode which tend to lead to shape inconsistencies. The shape inconsistencies directly reduce the alignment accuracy of the registered network, obviously.

**Table 1:** Quantitative comparison to state-of-the-arts on the Anti-UAV test set.

Networks	NCC$\uparrow$	SSIM$\uparrow$	HIST$\uparrow$	PSNR$\uparrow$	NMI$\uparrow$	MSE$\downarrow$
Misaligned input	1.5559	0.4788	0.2711	12.5179	0.1517	12971.34
DFMIR	1.5158	0.4691	0.2625	12.1195	0.1451	13675.71
VoxelMorph	1.4125	0.4573	0.2590	11.3597	0.1769	15908.30
SbR	1.5278	0.4658	0.2461	11.9921	0.1840	13739.73
NEMAR	1.5767	0.4808	0.2765	12.3069	0.1599	13343.28
UMF-CMGR	1.5531	0.4923	0.2784	12.5357	0.1898	12728.03
Our	1.5875	0.5144	0.2826	12.8942	0.1957	11880.82

All methods are re-trained on the Anti-UAV training set. Our method achieves the state-of-the-art under all six common evaluation metrics.

On the contrary, while other methods are as yet struggling with keeping the geometric information constant, our registration network successfully aligns images from different pairs of modalities and handles different alignment cases. It can be seen that our method is capable of accurately registration both small UAV targets and large building background. Our registration network can predict more accurate deformation fields, even when there exists significant shape deformation and style difference between source images and target images. This is mainly due to the following two points. First, the proposed ICMTN module reduces modal differences while preserving the original image geometry information which helps the registration module to better locate and align the targets. Second, the proposed MRDRN module captures detailed local texture information by modelling detailed image patches to drive alignment of small targets.

#### Component analysis

We sequentially inserted the MPTN and SCN into VoxelMorph and MRDRN as their improved versions, then adopt them to conduct cross-modality VIS-Infrared image registration. As shown in Table 2, their quantitative results improve a large margin in collaboration with MPTN and SCN than the original versions.

**Figure 8:**
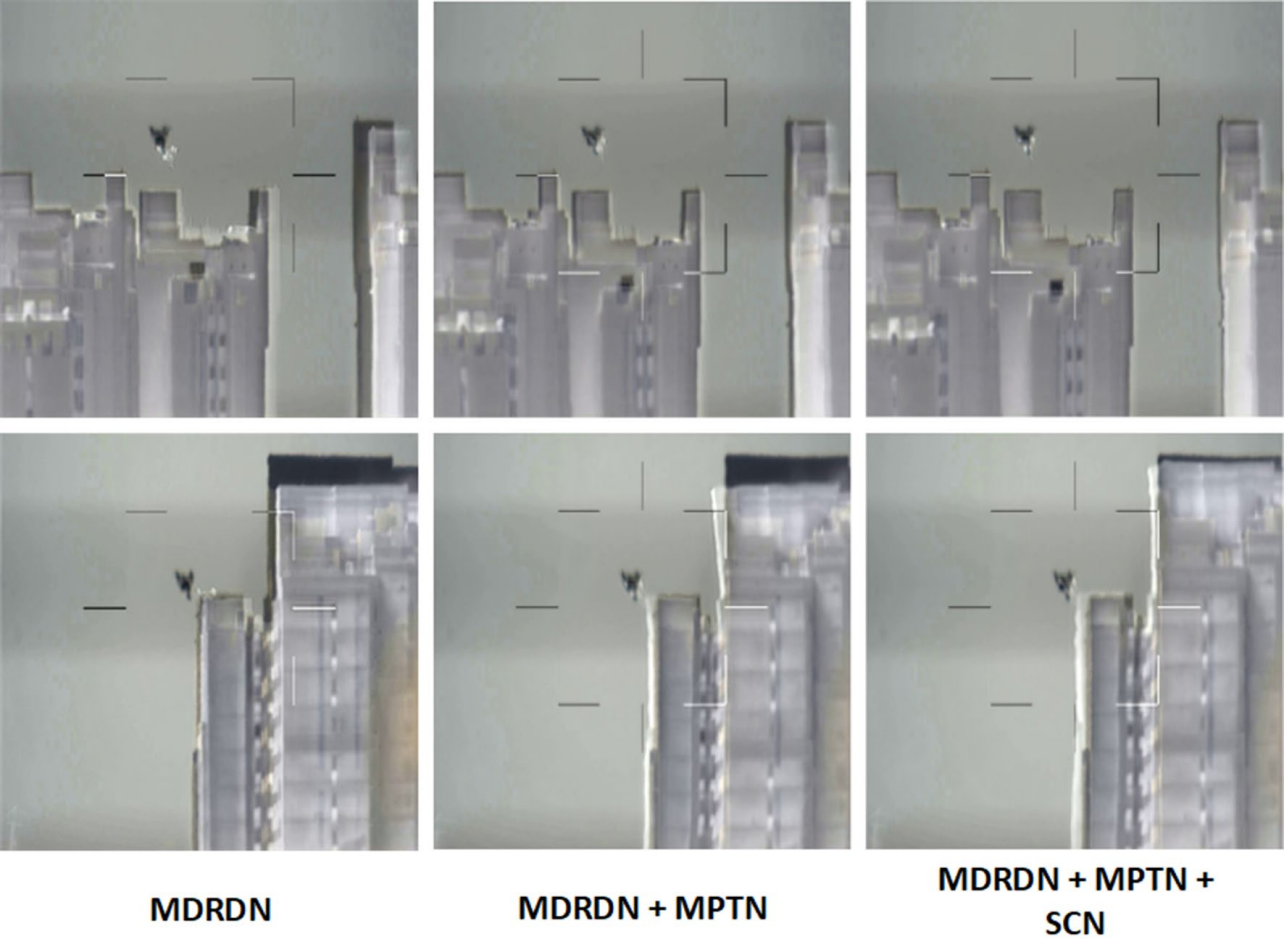
Ablation analysis of the MDRDN and MPTN on Anti-UAV datasets. Each row is a different experimental scenario. The first column shows the results of the MDRDN model, the second column shows the results of the MDRDN model using the MPTN, and the third column shows the results of the MDRDN model using both the MPTN and the SCN.

**Table 2:** Component analysis.

Networks	NCC$\uparrow$	SSIM$\uparrow$	HIST$\uparrow$	PSNR$\uparrow$	NMI$\uparrow$	MSE$\downarrow$
VoxelMorph	1.4125	0.4573	0.2590	11.3597	0.1769	15908.30
VoxelMorph + MPTN	1.5256	0.4771	0.2709	11.9761	0.1831	13492.27
VoxelMorph + MPTN + SCN	1.5684	0.4973	0.2791	12.6371	0.1914	12529.83
MRDRN	1.4674	0.4632	0.2655	11.7396	0.1817	14791.87
MRDRN + MPTN	1.5628	0.5073	0.2722	12.5117	0.1874	12744.31
MRDRN + MPTN + SCN	1.5875	0.5144	0.2826	12.8942	0.1957	11880.82

“VoxelMorph” and “MRDRN” refers to direct image registration without the use of modal translation networks. “MPTN” and “SCN” denote Modal Pre-Translation Network and Structure Correction Network.

Accordingly, the visual comparisons provided in Fig. 8 suggest the effectiveness of the MPTN and SCN. We observe that the registered results generated by the MRDRN model equipped with the MPTN and SCN eliminate evident distortion. The MPTN and SCN contributes to favorable fusion results with negligible ghosts for misaligned IR and VIS images. The above results comprehensively reveal the effectiveness of MPTN and SCN from registration and fusion perspectives.

## Conclusion

In this paper, we have proposed an important problem of Visible and Infrared UAV target Image Registration and provides an ideology for UAV target detection under multimodal data sources. A novel General Cross-Modality Registration Framework GCMR is also proposed to address this challenging task. It leverages Structure Correction Network (SCN) to ensures shape consistency while the Modal Pre-Translation Network (MPTN) enables the appearance transfer. Furthermore, it have engaged Multi-level Residual Dense Registration Network (MRDRN) with enhanced alignment performance, to predict the deformation field from coarse to fine between distorted and pseudo infrared images and reconstruct the registered infrared image. Extensive evaluations on the images in the Anti-UAV test set verify the effectiveness of our network.

## Data Availability

The datasets generated and analysed during the current study are not publicly available due Naval University of Engineering requirements but are available from the corresponding author on reasonable request.
